# One-Week Hydration Characteristics of Silica-Alumina Based Cementitious Materials Composed of Phosphorous Slag: Phosphorus Involved in Calcium Alumino-Silicate Hydrate Gel

**DOI:** 10.3390/ma18143360

**Published:** 2025-07-17

**Authors:** Zipei Li, Yu Wang, Jiale Zhang, Yipu Wang, Na Zhang, Xiaoming Liu, Yinming Sun

**Affiliations:** 1 School of Materials Science and Technology, China University of Geosciences (Beijing), Beijing 100083, China; 2 School of Metallurgical and Ecological Engineering, University of Science and Technology Beijing, Beijing 100083, China; 3 School of Medicine, University of California, San Diego, CA 92093, USA

**Keywords:** phosphorous slag, silica-alumina based cementitious materials, early-age hydration, microstructure

## Abstract

Phosphorous slag is an industrial by-product generated in the process of producing yellow phosphorus by electric furnace, which occupies a substantial number of land resources and causes serious environmental pollution. The comprehensive utilization of phosphorous slag is a major topic relevant to the sustainability of the yellow phosphorus industry. In this paper, we attempted to utilize phosphorous slag as a supplementary cementing material to prepare silica-aluminum based cementitious material (SAC-PHS). To determine how phosphorus influences the early-age hydration reaction process of silica-aluminum based cementitious material, three groups of samples, PHS_20_, PHS_25_, and PHS_30_, with better mechanical properties were selected to deeply investigate their one-week hydration characteristics. Characterization results showed that the main hydration products of SAC-PHS were C-A-S-H gels and ettringite. PHS_25_ specimen produced more C-A-S-H gels and ettringite than the other two samples after one-week hydration. Interestingly, the P/Si atomic ratio indicated that chemical bonds were formed between Si and P during the formation of C-A-S-H gels, which improved the strength of SAC-PHS. Our findings offer valuable insights for the application of phosphorous slag in construction and building materials and promote the efficient resource utilization of phosphorous residue.

## 1. Introduction

Phosphorous Slag (PHS) is an industrial byproduct generated during the production of yellow phosphorus by the electric furnace method, with approximately 10 tons of phosphorous slag produced for each ton of yellow phosphorus [1,2]. As a dominant player in the global arena of yellow phosphorus production, consumption, and export, China annually confronts over eight million tons of phosphorous slag emissions [3]. Paradoxically, the effective utilization rate of this slag remains disappointingly low, with merely 10% of the annual output being repurposed [4]. Consequently, vast quantities of phosphorous slag accumulate in open fields, commandeering extensive tracts of land [5]. Moreover, the phosphorous slag contains soluble phosphorous and fluorine compounds that can leach out by rainwater, resulting in pollution and environmental damage. Hence, the imperative to advance large-scale, resource-efficient utilization of phosphorous slag is paramount for fostering the sustainable progression of the yellow phosphorus industry and safeguarding our ecosystems.

Currently, numerous experts and scholars are dedicatedly exploring sustainable and circular applications for phosphorous slag, focusing on areas such as the development of glass-ceramics [6], the creation of decorative construction materials [7], the enhancement of ultra-high-performance concrete [8], and the retrieval of valuable resources [1]. These endeavors reflect a concerted effort towards realizing the full potential of phosphorous slag as a versatile and environmentally responsible resource. The shared characteristics of the phosphorous slag treatment methods include a low input-output ratio, weak market demand, and a complex processing methodology, all of which conspire to restrict the comprehensive utilization rate below 50%. An effective scheme should be proposed to treat phosphorous slag on a large scale to effectively address environmental challenges and improve economic efficiency. Employing solid waste as a supplementary cementitious material for the fabrication of gelatinous substances constitutes an efficacious strategy for the comprehensive utilization and valorization of waste resources. Supplementary cementitious materials [9] are generally composed of silica-alumina solid wastes, such as fly ash, ground Granulated Blast Furnace Slag (GBFS), coal gangue, red mud, etc., as the main raw material with a small amount of additives made of a variety of mineral compounds, prepared at room temperature and pressure, which are different from the traditional Portland cement (high-calcium system of cementitious materials) [10]. Carvalho et al. [11] reported that low-energy basic oxygen furnace slag could function effectively as both fillers and supplementary cementitious components, increasing the eco-efficiency of the cement-based composites regarding their mechanical performances. Wang et al. [12] investigated the potential of copper slag as a supplementary cementitious material. They collected various types of copper slag samples to establish reactivity indices and assess their safety, encompassing the stability of heavy metals, radioactivity, and leaching risks, which contributed to a deeper understanding of the application of copper slag in cementitious materials. Ramakrishnan et al. [13] investigated the impact of combining crushed waste glass powder and GBFS as partial replacements for cement on the mechanical properties and durability characteristics of concrete. A total of ten different mix proportions were studied at concrete ages of 3, 7, and 28 days, and the optimal blend was determined based on the compressive strengths of the various mixes at these ages. Their findings revealed that incorporating glass powder and GBFS reduced the permeability and water absorption rates of the concrete, thereby validating the successful application of crushed waste glass powder and GBFS as effective mineral admixtures in cementitious concrete.

As a product of high temperature quenching, phosphorous slag rapidly cools as expelled molten slag, resulting in a high glassy phase content of about 80–90% that imparts potential hydration activity [14]. The chemical composition and mineralogical composition of phosphorous slag is very similar to that of GBFS. The main chemical components of phosphorous slag are calcium oxide (35–50 wt%) and silica (35–45 wt%), so it has the potential to replace GBFS as a supplementary cementitious material [15]. Drawing upon extensive research on utilizing solid wastes as supplementary cementitious materials and considering the similarities between phosphorous slag and GBFS, this paper is guided by the isomorphism effect of the silica-alumina system on phosphorus for developing a kind of silica-alumina based cementitious material composed of phosphorous slag (SAC-PHS). Herein, the term “silica-alumina based cementitious material” (SAC) refers to a low-calcium binder system primarily composed of aluminosilicate-rich industrial by-products [16], including GBFS and fly ash, supplemented with a minimal proportion of Portland cement and desulfurization gypsum. Unlike traditional alkali-activated materials, SAC does not rely on highly alkaline activators such as sodium hydroxide or sodium silicate. It also differs from conventional blended cements by deliberately employing a SiO_2_-Al_2_O_3_-rich matrix to form C-A-S-H gel structures with low Ca/Si ratios, resulting in unique microstructural properties. This composition enhances early-age flexural strength and environmental performance compared to ordinary Portland cement systems [17,18].

Despite increasing interest in phosphorous slag as a supplementary cementitious material, most existing studies either depend on highly alkaline activators or examine PHS in isolation. Few investigations have explored its behavior in low-alkali, silica-alumina-based cementitious systems combined with other industrial by-products, such as GBFS and fly ash. Additionally, the influence of phosphorus on early-age hydration behaviors and microstructural evolution remains poorly understood.

To address these research gaps, this study examined the synergistic effects of phosphorous slag, GBFS, fly ash, and desulfurization gypsum in a low-alkali silica-alumina-based binder system. It indicated that incorporating phosphorous slag facilitates early-age gel formation through P-O-Si/Al bonding, thereby improving flexural performance and reducing environmental impact. The key novelty of this work lied in developing and validating a low-cement, low-alkali cementitious matrix incorporating phosphorous slag without requiring strong alkali activators. By preparing SAC-PHS and studying its one-week hydration products, the evolution of phosphorus was analyzed during the early-age hydration process. The investigation includes the impact of varying phosphorus dosages on the atomic ratios of P/Si, Al/Si, and Ca/Si in the hydrated cementitious products of SAC-PHS. Moreover, this study delved into how phosphorus doping affects the early-age microstructure of these hydration products, with the aim of achieving significant incorporation levels of phosphorous slag in cementitious materials. The research contributes to the understanding of the chemical interactions within the silica-alumina-phosphorus system and offers insights into optimizing the performance of cementitious composites containing phosphorous slag.

## 2. Experimental Section

### 2.1. Raw Materials

The raw materials selected for this experiment are phosphorous slag, ground granulated blast furnace slag, PII 52.5 Portland cement, fly ash, desulfurization gypsum, and standard sand. The origins of raw materials used in this research are shown in Table 1. Among them, the standard sand complied with the ISO national standard [19], and the particle sizes of GBFS, fly ash, and desulfurization gypsum were carefully controlled within the range of 200–300 mesh.

**Figure 1:**
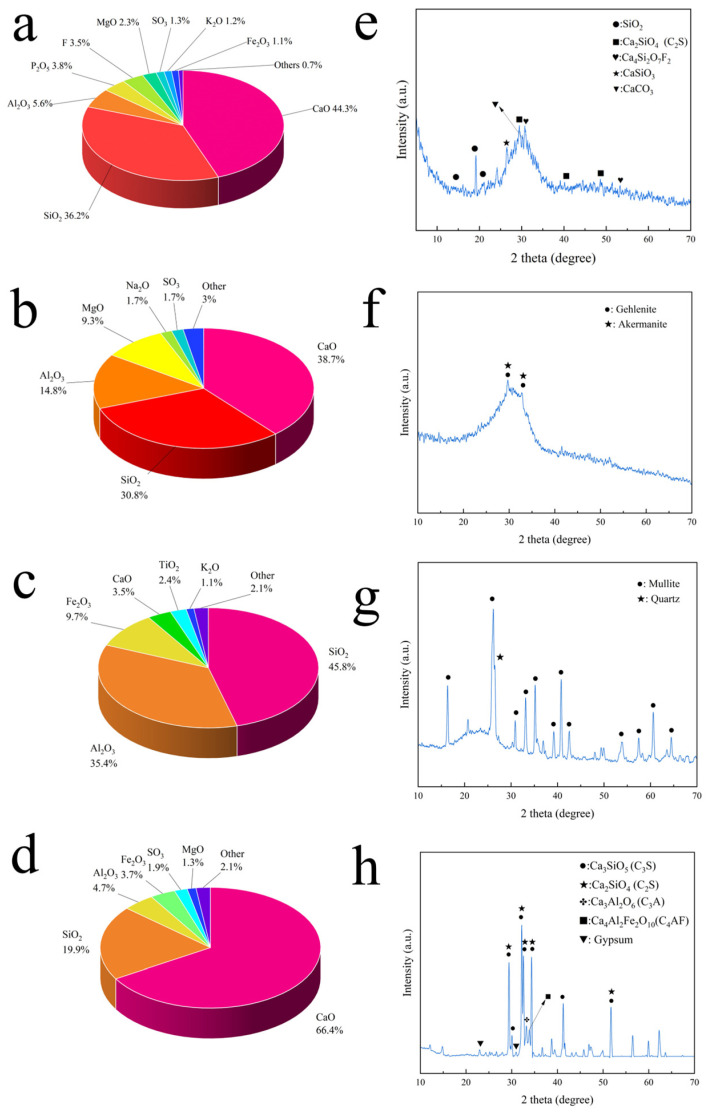
XRF analysis of phosphorous slag (**a**), GBFS (**b**), fly ash (**c**), and PII 52.5 Portland cement (**d**); XRD pattern of phosphorous slag (**e**), GBFS (**f**), fly ash (**g**), and PII 52.5 Portland cement (**h**).

The chemical composition of raw materials was analyzed using an XRF-1800 sequential X-ray fluorescence spectrometer (Shimadzu Corporation, Kyoto, Japan). As depicted in Figure 1a, the phosphorous slag primarily comprised calcium and silicon, with CaO and SiO_2_ collectively accounting for 80.5% by mass and a CaO/SiO_2_ ratio of 1.22. Notably, it also contained appreciable amounts of Al_2_O_3_, P_2_O_5_, MgO, K_2_O, SO_3_, Fe_2_O_3_, and fluoride compounds. According to XRF results, the P_2_O_5_ content in the phosphorous slag is 3.8%, which corresponds to a residual phosphorus (P) content exceeding 1.5% by mass. As depicted in Figure 1b, GBFS primarily comprised calcium, silicon, and aluminum oxides, with CaO, SiO_2_, and Al_2_O_3_ collectively accounting for 84.3% by mass. The CaO/SiO_2_ ratio was 1.26. Notably, it also contained significant amounts of MgO (9.3%) and minor components such as Na_2_O, SO_3_, and other oxides. Specifically, the Na_2_O and SO_3_ contents were both 1.7%, while other oxides constituted 3% of the total composition. The XRF results indicated that fly ash (Figure 1c) consists mainly of SiO_2_ (45.8%) and Al_2_O_3_ (35.4%), totaling 81.2 mass%. Fe_2_O_3_ represented the third major component at 9.7%. The remaining composition included CaO (3.5%), TiO_2_ (2.4%), K_2_O (1.1%), and other oxides (2.1%). The XRF analysis of PII 52.5 Portland cement (Figure 1d) revealed a dominant composition of CaO (66.4%) along with significant amounts of SiO_2_ (19.9%) and Al_2_O_3_ (4.7%), collectively making up 90% of the total mass. This cement exhibited a notably high CaO/SiO_2_ ratio of 3.34, reflecting its strong calcium-rich character. The composition further included Fe_2_O_3_ (3.7%) as the most prominent minor component, followed by SO_3_ (1.9%), MgO (1.3%), and trace amounts of others (2.1%).

Figure 1e illustrates the XRD analysis of phosphorous slag. The results showed that phosphorous slag confirmed a predominantly vitreous structure post rapid cooling [20], and detectable crystalline phases such as Ca_4_Si_2_O_7_F_2_, CaSiO_3_, C_2_S, CaCO_3_, and SiO_2_. Over 90% of the phosphorous slag exhibited a glassy matrix, with fluorine predominantly present in Ca_4_Si_2_O_7_F_2_ rather than in the crystalline form of Ca_5_(PO_4_)_3_F. This suggests that the fluoride in phosphorous slag exists in an insoluble form, having a negligible impact on the cementitious material’s setting time [20]. Figure 1f shows the XRD pattern of GBFS. It could be seen that the XRD results of GBFS and phosphorous slag both had rather disordered patterns. Meanwhile, GBFS included gehlenite and akermanite. The XRD results of fly ash and PII 52.5 Portland cement were clearer (Figure 1g,h). Fly ash obviously contained quartz and mullite. The main mineralogical components of PII 52.5 Portland cement were tricalcium silicate, dicalcium silicate, tricalcium aluminate, and tetralcium ferric aluminate. And it also contained a small amount of gypsum.

### 2.2. Preparation of Silica-Alumina Based Cementitious Materials Composed of Phosphorous Slag

**Figure 2:**
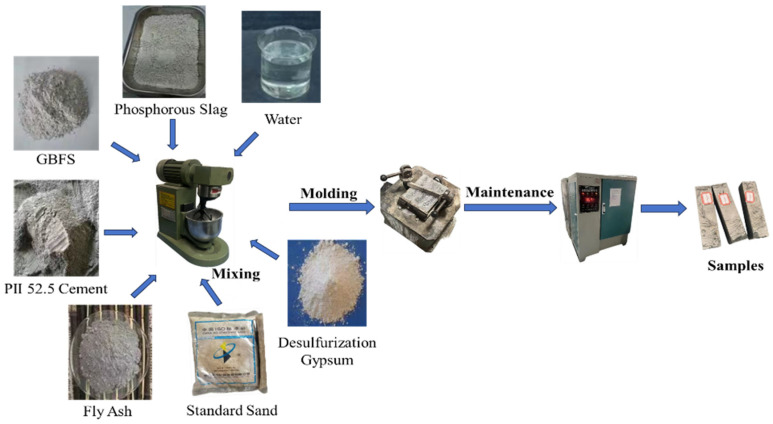
The preparation process of SAC-PHS mortars.

Mortar samples were prepared following methodologies outlined by GB/T 17671-2021 standard [21]. SAC-PHS mortars were fabricated with a water-to-binder ratio of 0.5, molded into 40 mm × 40 mm × 160 mm specimens, adhering to the experimental setup detailed in Table 2. The specific preparation steps of SAC-PHS mortars are illustrated in Figure 2. The pastes of SAC-PHS were prepared by removing standard sand in the preparation process of its mortar sample, according to a water-to-binder ratio of 0.35, molded into 20 mm × 20 mm specimens.

As displayed in Figure 2, the SAC-PHS mortar sample preparation procedure entailed several steps: (1) Raw materials were blended in an RSJ-5L mortar mixer at 250 rpm for 120 s. (2) Molds prepped with lubricating oil on their interiors were positioned on a vibrating table. (3) Post-mixing, the slurry was poured into the molds and vibrated for another 120 s, followed by excess removal and wrapping it in a preservative film to preserve moisture. (4) For curing, the molds spent 24 h in a DZF moist cabinet (Beijing Kewei Yongxing Instrument Co., Ltd., Beijing, China) set at 95% humidity and 20 °C, before being demolded and transferred to an isothermal curing cabinet (YH-40B, Sivaka Precision Gauge (Dongguan) Co., Dongguan, China) maintaining the same environmental conditions. Finally, after one-week hydration, the samples underwent flexural and compressive strength testing.

The procedure of preparing paste samples involved the following steps: (1) The mixer was set to standby mode, and the water was added to the paste mixing pot, followed by the raw materials. The pot was then placed on a fixed stand and raised to the designated position. (2) The mixer was started in automatic control mode, which included slow mixing for 120 s, a 15 s pause, and then fast mixing for another 120 s. (3) After mixing, the pot was removed, and the paste was layered into the molds (pre-coated with oil or release agent) using a spatula. The molds were placed on a vibrating table and compacted for 120 s. The molds were removed, and the surface of the samples was leveled with a scraper. (4) The molded paste specimens, along with their molds, were placed in a standard curing chamber maintained at a temperature of 20 °C and a relative humidity of no less than 90%. After 24 h of curing, the specimens were demolded.

### 2.3. Testing Methods

After one-week hydration, the flexural and compressive strengths of SAC-PHS mortars were measured. Flexural strength testing employed a three-point bending method at a loading pace of 50 N/s. The compressive strength test was carried out using a WAW-2000E electro-hydraulic servo hydraulic universal testing machine (Shandong Zhongyi Instrument Co., Ltd., Jinan, China).

SAC-PHS paste samples cured for one week were immersed in ethanol for 72 h to halt hydration, followed by vacuum drying at 55 °C for 12 h. After gold sputtering [22], scanning electron microscope (SEM) images were acquired, operated at 20 kV voltage and 10 mA current, to observe their microstructure of hydration products.

To further analyze the structure of SAC-PHS hydration products, powder samples were examined using X-ray diffraction (XRD) with settings of 40 kV voltage, 100 mA current, a scan speed of 8 °/min, and a scanning range from 5 ° to 70 ° using Cu Kα1 radiation. Next, infrared spectra were obtained for the SAC-PHS samples through the following procedure: a mixture of 5 mg powder sample and 200 mg KBr was homogenized, pressed into transparent pellets, and analyzed using an excitation wavelength of 785 nm.

Considering the pore structure’s significance in the hydrated paste, mercury intrusion porosimetry (MIP) was employed to investigate the pore characteristics of hydrated SAC-PHS pastes within a pressure range of 200 MPa. To ascertain the coordination environments of elements in the hydration products of SAC-PHS, samples underwent magic angle spinning nuclear magnetic resonance (MAS-NMR) at room temperature, with a rotational speed of 12 kHz. Specifically, NMR spectra for one-week hydration products were collected individually for Al, P, and Si elements.

## 3. Results and Discussion

### 3.1. One-Week Strength of Silica-Alumina Based Cementitious Materials Composed of Phosphorous Slag

**Figure 3:**
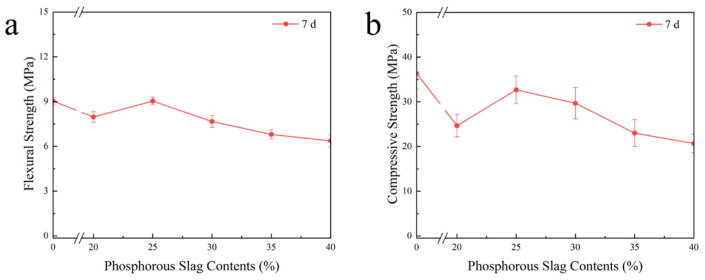
The flexural strength (**a**) and compressive strength (**b**) of SAC-PHS after one-week hydration.

Based on the composition of raw materials and the distribution ratio presented in Table 2 for SAC-PHS, Figure 3a depicts the flexural strength of SAC-PHS after one-week hydration. The 7-day flexural strength of SAC-PHS decreased as the phosphorous slag content rose yet peaked at 9.03 MPa when doped with 25% phosphorous slag, surpassing the flexural strength of a non-doped sample (9.00 MPa). Notably, SAC-PHS maintained considerable flexural strength even with 30% phosphorous slag content.

Figure 3b illustrates the compressive strength of SAC-PHS after one week hydration, exhibiting a parabolic trend with the increase in phosphorous slag content. Like 7-day flexural strength, a peak in 7-day compressive strength was observed with 25% phosphorous slag content, whereas a marked decline occurred at 35% doping, suggesting that increased phosphorous slag dosage did not universally enhance performance [23]. Consequently, for the further experiments focusing on hydration products and microstructure, three formulations, PHS_20_, PHS_25_, and PHS_30_, representing higher strength levels were selected.

**Figure 4:**
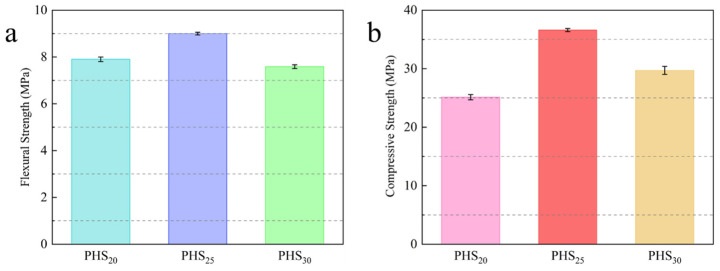
Comparison of 7-day flexural strength (**a**) and compressive strength (**b**) among PHS_20_, PHS_25_, and PHS_30_ (mean ± SD, *n* = 5).

To assess the statistical significance of the one-week mechanical strength differences between the PHS_20_, PHS_25_, and PHS_30_ formulations (Figure 4), a one-way analysis of variance (ANOVA) was performed [24,25]. The ANOVA results demonstrated statistically significant variations in both flexural strength (*p* < 0.05) and compressive strength (*p* < 0.01), indicating that the observed performance differences were systematic rather than random. Subsequent post hoc analysis using Tukey’s Honestly Significant Difference (HSD) test [26] identified that PHS_25_ achieved significantly superior mechanical strength compared to both PHS_20_ and PHS_30_. These statistical findings validate the selection of PHS_25_ as the optimal formulation for early-age mechanical performance of SAC-PHS.

**Figure 5:**
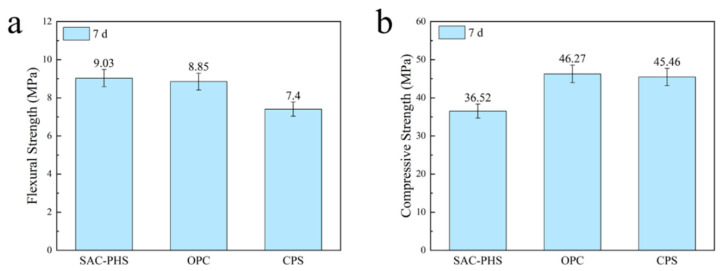
The flexural strength (**a**) and compressive strength (**b**) of different cementitious materials after one-week hydration.

Figure 5 presents comparisons of the one-week hydration characteristics in terms of flexural and compressive strengths, which were made between SAC-PHS (with 25% phosphorous slag content), ordinary Portland cement (OPC) [27,28], and Portland cement solely doped with phosphorous slag (CPS) (with 25% phosphorous slag content) [29,30].

After one-week hydration, the flexural strength of SAC-PHS reached 9.03 MPa, which was 2% higher than that of OPC and 22% higher than that of CPS. The experimental results show that SAC-PHS exhibits excellent flexural strength after a hydration time of one week. Its flexural strength not only exceeded OPC but can also effectively avoid the influence of phosphorus incorporation on the strength of Portland cement. However, after one-week hydration, the compressive strength of SAC-PHS reached 36.52 MPa, which decreased by 21% compared with OPC and 19% compared with CPS. This demonstrates that SAC-PHS displays a marked reduction in compressive strength relative to OPC. Adding phosphorous slag to Portland cement caused a certain negative effect on its compressive strength.

According to the changing laws of compressive and flexural strengths of SAC-PHS, OPC, and CPS, it should be known that adding phosphorous slag into ordinary Portland cement had a negative effect on its early-age flexural strength. SAC-PHS had a great performance in flexural strength and poor performance in compressive strength after one-week hydration. On account of flexural strength’s sensitivity to microcracking [31], the generation of microcracks can break down the stress on the material and have a positive effect on the flexural strength of the material. Therefore, the silica-alumina system may improve the pore structure of the mortar and increase the generation of micro-cracks, enhancing its density and the interface bond between the aggregate and the paste. This, in turn, bolstered the flexural strength [32]. However, the generation of micro-cracks has a negative impact on the compressive strength of the material [33], which also explains why the compressive strength of SAC-PHS is lower than that of OPC and CPS.

In recent years, researchers made various attempts to improve the reactivity of phosphorous slag in cementitious systems, aiming to enhance early strength. Referring to Wang et al. [34], cementitious materials were also prepared using phosphorous slag and fly ash, and the compressive strength of the sample PSC3 prepared by them was about 33 MPa after a week of hydration time. He et al. [23] prepared eco-cement mortar containing MgO-modified phosphorous slag; the samples’ compressive strength after one-week hydration were all lower than 30 MPa. Experimental results indicate that the SAC-PHS developed in this study exhibited superior mechanical properties after one-week hydration compared to other cementitious materials incorporating phosphorous slag.

### 3.2. One-Week Hydration Characteristics of Silica-Alumina Based Cementitious Materials Composed of Phosphorous Slag

Given the flexural strength outcomes, which highlighted improved mechanical properties in silica-alumina cementitious materials with phosphorous slag dosages of 20%, 25%, and 30%, the decision was made to focus on the analysis of hydration characteristics on the PHS_20_, PHS_25_, and PHS_30_ formulations.

#### 3.2.1. SEM-EDS Analysis

**Figure 6:**
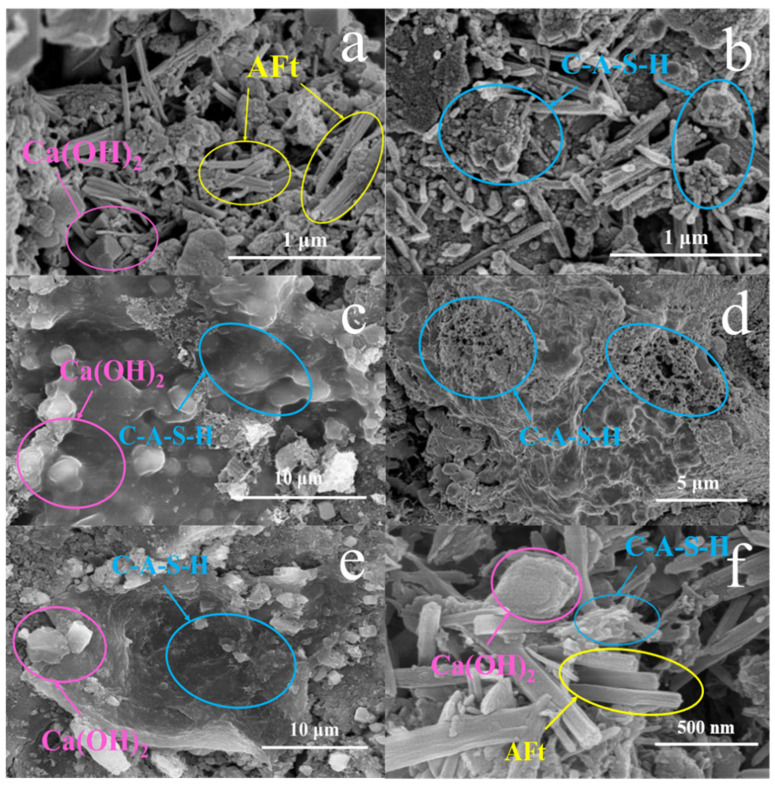
SEM images of SAC-PHS with 20% (**a**,**b**), 25% (**c**,**d**), and 30% (**e**,**f**) phosphorous slag content after one-week hydration.

Figure 6a and 6b reveal an abundance of acicular ettringite (AFt) [35,36] and flocculent C-A-S-H gels [37] after one week of hydration, respectively. The gel network bound the ettringite crystals, densifying the paste and bolstering the hardened material’s strength. Notably, Figure 6a reveals Ca(OH)_2_ encapsulated in gels, suggesting the partial retention of unreacted Ca(OH)_2_ in its original form within PHS_20_. As Figure 6c,d depict, many Ca(OH)_2_ blocks had disappeared, and those remaining were encircled by a gel-like substance. This evidence attests that, in the 7-day hydration products, Ca(OH)_2_ progressively converted into AFt, accompanied by the generation of C-A-S-H gels. Simultaneously, the flocculent gels transitioned into 3-dimensional mesh structures, which diminished pore spaces between particles and fortified the material’s strength. Figure 6e,f depict the 7-day hydration product of PHS_30_, illustrating abundant C-A-S-H gels with a 3-dimensional mesh structure surrounding SiO_2_ and Ca(OH)_2_, alongside numerous needle-shaped AFt crystals. These observations confirmed a substantial enhancement in PHS_30_’s strength following the 7-day hydration period.

**Figure 7:**
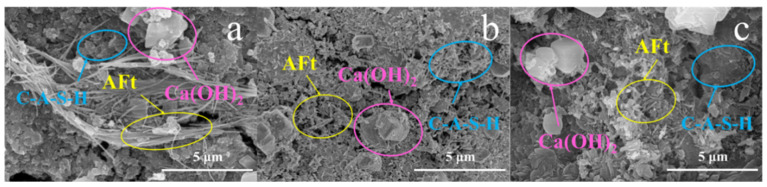
SEM images of PHS_20_ (**a**), PHS_25_ (**b**), and PHS_30_ (**c**) after one-week hydration.

Comparing the hydration products of PHS_20_, PHS_25_, and PHS_30_ after a one-week period, Figure 7 reveal the following fact. At the one-week hydration mark, all three samples of PHS_20_, PHS_25_, and PHS_30_ exhibited conspicuous C-A-S-H gels and abundant needle-like ettringite. Notably, C-A-S-H gels in PHS_20_ were clustered and devoid of a 3-dimensional mesh structure, suggesting that phosphorous slag inclusion facilitated gel formation to some extent. A marked increase in Ca(OH)_2_ was observed in PHS_30_ compared to PHS_25_, implying that the promotional influence of phosphorous slag on SAC-PHS hydration was non-linear and peaking at a specific content. This finding aligns with the mechanical properties’ experimental outcomes for SAC-PHS, highlighting the beneficial impact of gel formation on enhancing SAC-PHS’s mechanical attributes.

**Figure 8:**
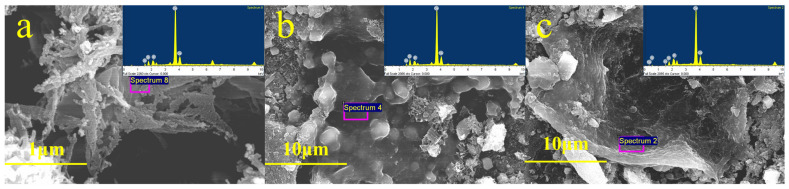
EDS analysis results of PHS_20_ (**a**), PHS_25_ (**b**), and PHS_30_ (**c**) after one-week hydration.

Energy dispersive spectra (EDS) were acquired from the C-A-S-H gels formed in one-week hydrated pastes of PHS_20_, PHS_25_, and PHS_30_, with analysis sites depicted in Figure 8. EDS analysis results revealed the presence of Al and P in these C-A-S-H gels. After one-week hydration, the calculated atomic ratios were as follows: PHS_20_ had Al/Si = 0.35, Ca/Si = 13.71, and P/Si = 0.17. PHS_25_ had Al/Si = 0.29, Ca/Si = 10.58, and P/Si = 0.11. PHS_30_ had Al/Si = 0.18, Ca/Si = 15.82, and P/Si = 0.23. These ratios reflect the elemental composition within the gels [38], furthering the understanding of the hydration process in these SAC-PHS materials.

The one-week hydrated pastes of PHS_20_, PHS_25_, and PHS_30_ exhibited P/Si atomic ratios of 0.17, 0.11, and 0.18, respectively, with Ca/Si atomic ratios of 13.71, 10.58, and 15.82. PHS_25_ displayed notably lower P/Si and Ca/Si atomic ratios compared to the other formulations. High Ca/Si ratios can result in excess free Ca(OH)_2_, detracting from material strength, whereas lower Ca/Si ratios potentially enhance bending resistance through increased C-A-S-H gel formation [39]. A reduced P/Si atomic ratio signifies higher silicon content; also the ratio showed that during the formation of C-A-S-H gels, a bond was formed between Si and P, facilitating more C-A-S-H gel production, which was advantageous for improving both early and late strength in silica-alumina cementitious materials [40,41]. These findings correlate with the outcomes of SAC-PHS’s mechanical tests and SEM analyses, affirming the positive impact on material performance.

The non-monotonic relationship between the P/Si atomic ratio and mechanical strength observed in this study can be explained from both thermodynamic and kinetic perspectives. At moderate phosphorus levels (e.g., PHS_25_), phosphate ions can be incorporated into the aluminosilicate network via P-O-Si or P-O-Al bonds, enhancing the cross-linking density and the degree of polymerization in C-A-S-H gels, thereby contributing to matrix densification and mechanical strength [42,43]. However, when the P/Si ratio becomes excessively high (e.g., PHS_30_), this beneficial effect may be offset by the formation of phosphate-rich secondary phases such as calcium phosphate or aluminum phosphate, which are thermodynamically stable but do not participate in strength-contributing gel networks [44]. Furthermore, excess phosphate may kinetically hinder the dissolution of aluminosilicate precursors by competing with silicate and aluminate species for Ca^2+^ or Na^+^ ions, thereby inhibiting gel growth or altering gel morphology [45]. These combined effects can lead to reduced gel volume and a more porous microstructure, which ultimately compromises compressive strength, despite a higher P content. This mechanism is consistent with earlier studies on phosphate-modified geopolymer and alkali-activated systems [46,47].

#### 3.2.2. XRD Analysis

**Figure 9:**
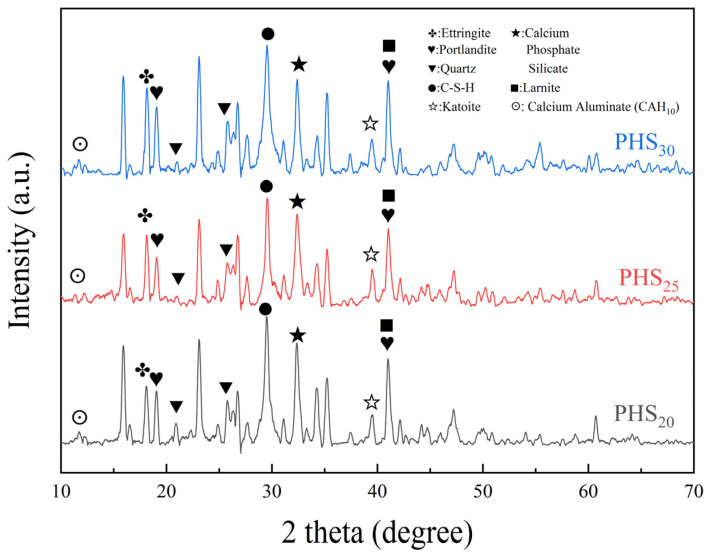
XRD patterns of SAC-PHS after one-week hydration.

Figure 9 shows the XRD patterns of PHS_20_, PHS_25_, and PHS_30_ after one-week hydration. Observation of the figure reveals that the principal hydration products of SAC-PHS comprise C-S-H gels, ettringite [48], calcium phosphate silicate [49], katoite, and calcium aluminate (CAH_10_) [50]. The production of calcium phosphate silicate indicates the main occurrence state of P element in SAC-PHS. Notably, the XRD pattern exhibits a wide, diffuse background signal between 25° and 38° 2 theta [51], indicative of the presence of amorphous C–A–S–H gels within the hydrated SAC-PHS pastes.

After one-week hydration, it was noted that Portlandite (Ca(OH)_2_) [52], Quartz (SiO_2_) [53], and Larnite (Ca_2_SiO_4_) [54] remained present in SAC-PHS. Given the inclusion of PII 52.5 cement in the mixture, high levels of Ca_2_SiO_4_ (C_2_S) and Ca_3_SiO_5_ (C_3_S) were initially present. However, analysis of the XRD data following one week of hydration showed clear evidence of consumption for both compounds, with C_3_S consumption so extensive that it was no longer detectable by XRD. Ca(OH)_2_, a crystalline byproduct primarily resulting from clinker hydration, undergoes consumption through pozzolanic reactions with phosphorous slag, fly ash, and GBFS. These reactions give rise to the formation of C–A–S–H gels, summarized by the subsequent expressions [55,56].

SiO_2_ + OH^−^ + H_2_O → [H_3_SiO_4_]^−^ (1)

AlO_2_^−^ + OH^−^ + H_2_O → [H_3_AlO_4_]^2−^ (2)

[H_3_SiO_4_]^−^ + [H_3_AlO_4_]^2−^ + Ca^2+^ → C-A-S-H (3)

Here, SiO_2_ signifies the reactive silica compounds present in phosphorous slag, fly ash, and GBFS, while AlO_2_^−^ denotes the reactive alumina substances dissolved from these components.

Comparative analysis of XRD patterns revealed diminished peaks for Ca(OH)_2_, SiO_2_, and Ca_2_SiO_4_ in PHS_25_ relative to PHS_20_ and PHS_30_. This suggests heightened participation of raw materials in PHS_25_’s hydration process, fostering enhanced C-A-S-H gels and ettringite formation, thereby boosting the material’s mechanical attributes.

#### 3.2.3. FTIR Analysis

**Figure 10:**
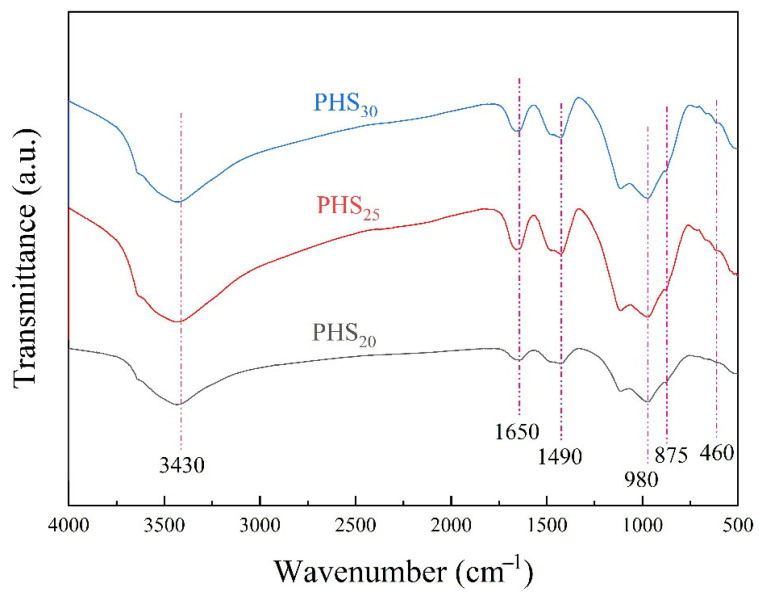
FTIR spectra of SAC-PHS after one-week hydration.

Figure 10 shows the FTIR spectra of the PHS_20_, PHS_25_, and PHS_30_ after one-week hydration. The infrared spectrum featured absorption bands at approximately 3430 cm^−1^ and 1650 cm^−1^, attributed to OH^−^ vibrations [57]. Bands around 1490 cm^−1^ signified the presence of CO_3_^2−^ [58]. The region around 980 cm^−1^ corresponded to the vibration of Si-O bond, whereas 875 cm^−1^ denoted the stretching vibration of SiO_4_^4−^ [59,60]. Lastly, a peak at roughly 460 cm^−1^ represented the characteristic band for Si-O-Si (Al) bending [61].

As shown in Figure 10, the stretching vibration band at circa 875 cm^−1^ displayed notable sharpening, suggesting a high degree of [SiO_4_] tetrahedra polymerization within the system. Comparison of PHS_20_, PHS_25_, and PHS_30_ samples revealed that PHS_25_ exhibited significantly increased absorption bands at 3430 cm^−1^ and 1650 cm^−1^ compared to PHS_20_ and PHS_30_. This implies a greater conversion of free water in PHS_25_ into bound water post-reaction engagement. Furthermore, the aggrandizing of the 980 cm^−1^ characteristic band signified increased Si-O bond breakage in PHS_25_ throughout the reaction. The stronger absorption at 460 cm^−1^ in PHS_25_ signified a more substantial breakdown of the glassy structure from phosphorus slag and GBFS powder during hydration, leading to a higher fraction of Si-O-Si and Al-O-Al bonds cleavage and integration into the hydrating medium than in PHS_20_ and PHS_30_. Collectively, these findings denote enhanced C-A-S-H gels formation in PHS_25_, conferring favorable mechanical properties to the material.

#### 3.2.4. MIP Analysis

**Figure 11:**
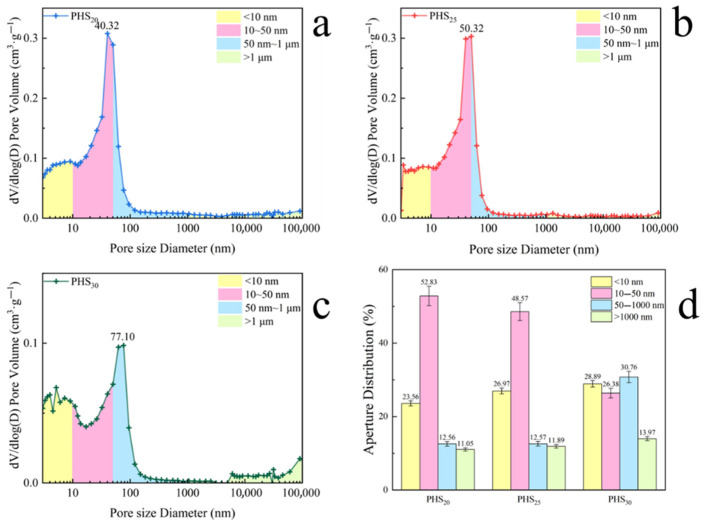
The most probable pore diameter of PHS_20_ (**a**), PHS_25_ (**b**), and PHS_30_ (**c**) after one-week hydration, and the different aperture distributions of PHS_20_, PHS_25_, and PHS_30_ after one-week hydration (**d**).

Figure 11a, b and c show that the most probable pore diameter of PHS_20_, PHS_25_, and PHS_30_ pastes after one-week hydration are 40.32 nm, 50.32 nm, and 77.10 nm, respectively. The pore diameters of PHS_20_ and PHS_25_ were basically concentrated below 50 nm, while that of PHS_30_ was concentrated below 100 nm. It is noted that the pore diameter increased with the increasing content of phosphorous slag. It had obvious disadvantages when the content of phosphorous slag reached 30%.

Figure 11d shows the different aperture distributions of PHS_20_, PHS_25_, and PHS_30_ after one-week hydration. Observation of the figure indicates that the pore diameters of PHS_20_ and PHS_25_ were also predominantly below 50 nm, with a notable concentration within the 10–50 nm range. Referring to the analysis of MIP by Du et al. [62], the PHS_30_ presented the largest proportion of gel pores (<10 nm) and the harmful macropores (>50 nm). Excessive harmful macropores seriously reduced the compressive strength of the material [63]. Conversely, PHS_30_ exhibited a broader pore size distribution, particularly showing a substantial increase in pores measuring 50–1000 nm compared to PHS_20_ and PHS_25_. Corroborated by XRD, and FTIR analyses, it is inferred that while PHS_30_ contained a fair number of C-A-S-H gels and a notable proportion of pores under 10 nm, its mechanical properties demonstrated a conspicuous decline relative to PHS_25_. This decline was attributed to the irregular expansion of C-A-S-H gels during PHS_30_’s hydration process, leading to a proliferation of larger pores that negatively impacted the material’s mechanical integrity [64].

As evidenced by the MIP results, PHS_30_ demonstrated higher total porosity compared to both PHS_25_ and PHS_20_, correlating well with its inferior compressive strength performance. The increased cumulative intrusion volume further revealed more extensive interconnected pore networks in PHS_30_, indicative of a less dense microstructure. Analysis of the most-probable pore diameter distribution showed that PHS_30_ exhibited a distinct peak at 77.10 nm, substantially larger than the 50.32 nm observed for PHS_25_. This shift in pore size distribution was accompanied by a marked reduction in mesopores (10–50 nm) and a corresponding increase in larger pores (>100 nm), including macropores. These microstructural characteristics collectively contributed to the compromised mechanical performance of PHS_30_. The coarser pore structure, characterized by both larger dominant pore sizes and enhanced pore connectivity, resulted in a more porous and less cohesive matrix. This dual microstructural effect provides a clear explanation for the reduced compressive strength of PHS_30._

#### 3.2.5. MAS-NMR Analysis

**Figure 12:**
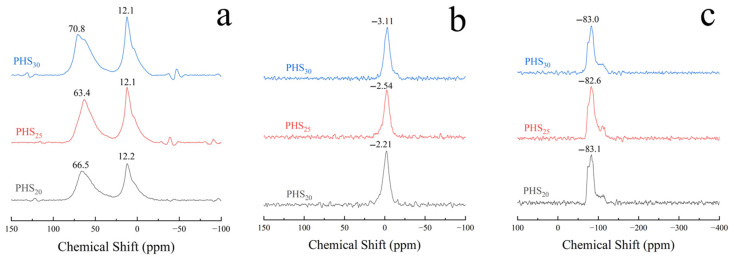
^27^Al-NMR (**a**), ^31^P-NMR (**b**), and ^29^Si-NMR (**c**) of SAC-PHS after one-week hydration.

Figure 12a depicts the ^27^Al-NMR spectra obtained from PHS_20_, PHS_25_, and PHS_30_ after one-week hydration. Each spectrum featured resonance peaks indicative of six-coordinate aluminum at a chemical shift of 12 ppm, presumably arising from [AlO_6_] octahedra [65,66]. A peak at 66.5 ppm was attributed predominantly to tetra-coordinate aluminum [67]. There was an overlapping peak around 70 ppm, which represented the combination of tetra-coordinated aluminum in the C-A-S-H gel and in the raw materials. Meanwhile, a peak close to 60 ppm signified unreacted tetra-coordinate aluminum.

Figure 12b illustrates the ^31^P-NMR spectra for samples PHS_20_, PHS_25_, and PHS_30_ after one-week hydration. The spectra revealed peaks occurring within the range of −2 to −3 ppm, suggesting that phosphate existed in a consistent structural configuration across these samples [68]. Figure 12c displays the ^29^Si-NMR spectra for PHS_20_, PHS_25_, and PHS_30_ following a one-week hydration period. Resonance peaks situated at approximately −83 ppm and −110 ppm signified SiQ^2^(1Al) and SiQ^4^ configurations, respectively, with the peak at −83 ppm exhibiting greater intensity [69,70]. This observation implied that SiQ^2^(1Al) constituted the predominant structural unit in the hydrated pastes of SAC-PHS at the one-week hydration mark.

To better interpret the MAS-NMR results of hydrated SAC-PHS pastes, it is important to consider the NMR spectral characteristics of the raw materials. According to the literature [71,72], phosphorous slag and GBFS typically exhibit broad ^27^Al signals centered around 60–70 ppm (tetrahedral Al) and weak signals near 9–15 ppm (octahedral Al), attributed to Al in amorphous phases. Portland cement generally shows intense Al^VI^ signals at 10–12 ppm and Al^IV^ signals at 60–70 ppm. Meanwhile, since the Portland cement has not undergone hydration, the signal of Al^IV^ is much stronger than that of Al^VI^ [73]. Fly ash usually contains both Al^IV^ (55–65 ppm) and Al^VI^ components [74]. These spectral features serve as references for distinguishing the hydration-related evolution of Al environments in SAC-PHS. Similar reference trends are also observed in ^29^Si MAS-NMR, where GBFS and fly ash are dominated by Q^0^-Q^2^ silicate species, while Portland cement shows signals in the Q^0^ region due to unreacted C_3_S/C_2_S [72,73,74].

**Figure 13:**
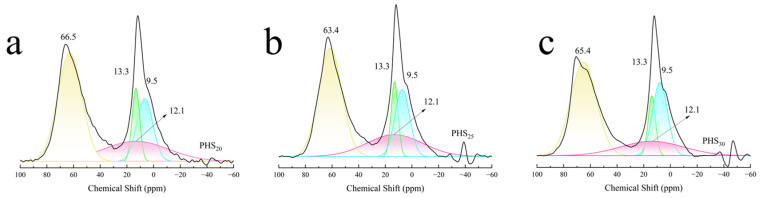
Deconvolution of ^27^Al-NMR spectra of PHS_20_ (**a**), PHS_25_ (**b**), and PHS_30_ (**c**) after one-week hydration.

Considering various contributions such as quadrupolar effects, we used the Czjzek model [75] for deconvolution of ^27^Al-NMR. The ^27^Al MAS-NMR spectra of the PHS_20_, PHS_25_, and PHS_30_ samples are shown in Figure 13. It was observed that the resonance peaks at chemical shifts of 66.5 ppm, 63.4 ppm, and 65.4 ppm were attributed to Al^IV^, while the resonance peaks at chemical shifts ranging from 9.5 ppm to 13.3 ppm were assigned to Al^VI^. Noticeably, Al^VI^ included many different aluminates hydrate phases where ettringite generates a signal at 13.3 ppm, katoite (C_3_AH_6_) at 12.1 ppm, and CAH_10_ at 9.5 ppm [50]. The analysis results of the ^27^Al-NMR spectra for PHS_20_, PHS_25_, and PHS_30_ are presented in Table 3. In the SAC-PHS system, aluminum existed in the form of both Al^IV^ and Al^VI^, with Al^IV^ being the dominant species. Compared to PHS_20_ and PHS_30_, the relative content of Al^VI^ in PHS_25_ significantly increased, while that of Al^IV^ decreased, suggesting the transformation of Al^IV^ to Al^VI^. It is thought that PHS_25_ produced more ettringite, katoite, and calcium aluminate hydrate during the early hydration process.

**Figure 14:**
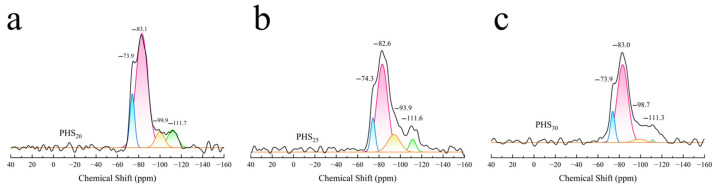
Deconvolution of ^29^Si-NMR spectra of PHS_20_ (**a**), PHS_25_ (**b**), and PHS_30_ (**c**) after one-week hydration.

Figure 14 shows the deconvolution of ^29^Si-NMR spectra of PHS_20_, PHS_25_, and PHS_30_ samples. As could be seen from the figure, after one week of hydration, the ^29^Si-NMR spectra of the samples mainly contained four resonance peaks, which belonged to the SiQ^0^ structure with chemical shifts of −73.9 ppm and −74.3 ppm, the SiQ^2^(1Al) structure with chemical shifts of −83.1 ppm, −82.6 ppm, and −83.0 ppm, the SiQ^3^ structure with chemical shifts of −99.9 ppm, −93.9 ppm, and −98.7 ppm, and the SiQ^4^ structure with chemical shifts of −111.7 ppm, −111.6 ppm, and −111.3 ppm. The gel products were mainly composed of SiQ^2^(1Al) structure.

The ^29^Si-NMR spectra of PHS_20_, PHS_25_, and PHS_30_ samples were subjected to peak fitting analysis. In ^29^Si-NMR, the number of relative bridging oxygen bonds is represented by SiQ*_n_* (*n* = 0–4). Zhang [76] discovered that the degree of polymerization of the silicon-oxygen tetrahedral structure [SiO_4_] could be quantitatively calculated by the amount of relative bridging oxygen (RBO). The calculation formula for the RBO is shown in Equation (4):

(4) $$RBO=\frac{1}{4}(1\times \frac{Q^{1}}{\sum Q^{n}}+2\times \frac{Q^{2}}{\sum Q^{n}}+3\times \frac{Q^{3}}{\sum Q^{n}}+4\times \frac{Q^{4}}{\sum Q^{n}})=\frac{1}{4}\frac{\sum nQ^{n}}{\sum Q^{n}}$$

where Q*^n^* is the relative peak area of the ^29^Si-NMR with RBO number *n*.

The RBO values obtained by calculating are shown in Table 4. It revealed that the polymerization degree of [SiO_4_] tetrahedra in SAC-PHS reached its maximum in the PHS_25_ sample. In accordance with the established principles governing [SiO_4_] tetrahedral polymerization (as described in Reference [77]), the incorporation of phosphorous slag was found to enhance the cementitious activity of silicon-aluminum based materials. As the phosphorous slag content increased, the polymerization degree of [SiO_4_] tetrahedra initially increased. However, excessive phosphorous slag content resulted in a subsequent decrease in the polymerization of PHS_30_.

The NMR analysis highlighted that the primary hydration products of the SAC-PHS comprised C-A-S-H gels and AFt, both contributing positively to strength development [62]. Aluminum predominantly existed in the hydrated products as tetra- and hexa-coordinated species, with hexa-coordination being prevalent. This dominance arose from the involvement of tetra-coordinated Al from raw materials in the hydration reactions. Consequently, [AlO_4_] tetrahedra transformed into [AlO_6_] octahedra within AFt crystal formation or combined with [SiO_4_] tetrahedra to form C-A-S-H gels. As the hydration progressed over time, the degree of [SiO_4_] polymerization intensified, culminating in the one-week hydration product predominantly assuming the configuration of SiQ^2^(1Al). Combining the above SEM-EDS and NMR analysis results, the presence of phosphorus (P) in the C-A-S-H gel suggested an isomorphic substitution mechanism where P replaces Si, analogous to the known Al substitution. This observation confirmed that a four-coordinated isomorphic substitution of Si by P occurs during the formation of C-A-S-H gel in the hydration process.

### 3.3. Carbon Emission and Economic Cost of Silica-Alumina Based Cementitious Materials Composed of Phosphorous Slag

To evaluate the sustainability and economic feasibility of the SAC-PHS system, a comparison was conducted between the optimized mixture (PHS_25_), the PHS_0_ control group, and 42.5 Portland cement, which is representative of the conventional ordinary Portland cement (OPC) system. The comparison focused on both embodied carbon emissions and material cost.

Embodied carbon is an important parameter to evaluate the environmental impact of silica-aluminum based cementing materials. The embodied carbon data of each raw material were shown in Table 5, which were calculated based on the “cradle-to-gate” system [78,79,80]. Herein, 42.5 Portland cement was adopted as the reference sample due to the compressive strength of the PHS_25_ exceeding 30 MPa after 7 days of hydration. Meanwhile, the total embodied carbon of the silica-alumina based cementitious material without the addition of phosphorous slag (PHS_0_) was also compared.

**Figure 15:**
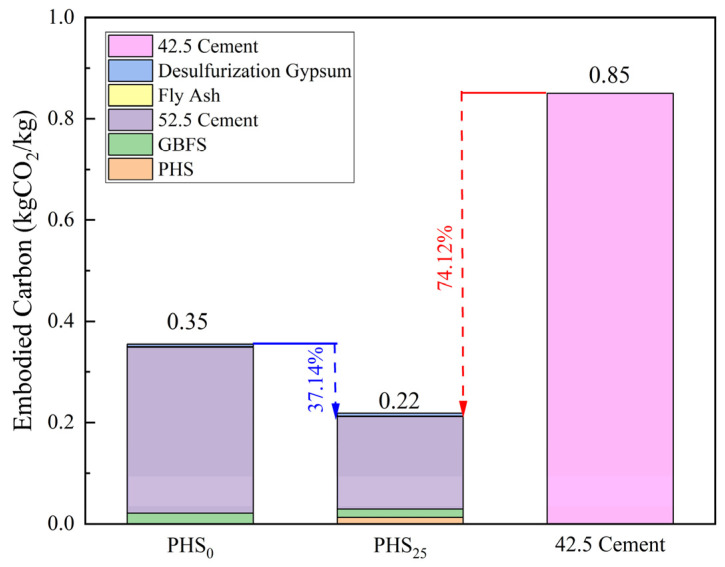
The embodied carbon comparison of PHS_0_, PHS_25_, and 42.5 cement.

The embodied carbon generated from the production of 1 kg of PHS_0_ and PHS_25_ was calculated, respectively, through experimental proportioning. As shown in Figure 15, the embodied carbon of PHS_25_ was 0.22 kgCO_2_/kg, which is 74.12% lower than the embodied carbon of 42.5 Portland cement. Compared with PHS_0_, PHS_25_ had reduced its embodied carbon by 37.14% due to the incorporation of low-carbon-emission PHS to replace part of the PII 52.5 cement. It is noteworthy that the PII 52.5 cement used for alkali excitation accounts for the largest proportion of the implicit carbon in PHS_25_ and PHS_0_, reaching 81.8% and 91.4%, respectively, while other solid waste materials account for a small proportion of less than 20%. Therefore, the silica-alumina based cementitious material is more conducive to the development concept of the low-carbon economy than the traditional high-calcium cement-based building materials.

**Figure 16:**
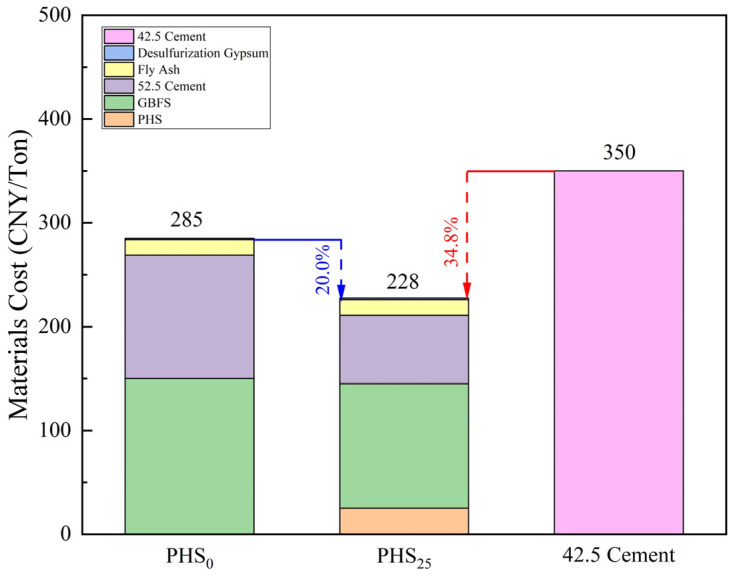
Material cost comparison of PHS_0_, PHS_25_, and 42.5 cement.

Figure 16 presents a comparative analysis of material costs among PHS_0_, PHS_25_, and conventional 42.5 Portland cement. Unlike traditional cementitious systems that depend primarily on expensive clinker-based cement, the SAC-PHS system utilized industrial by-products—including GBFS, fly ash, and desulfurization gypsum, resulting in substantially lower material costs. Quantitative evaluation revealed that PHS_25_ had a total cost of 228 CNY/Ton, which was 20.0% lower than PHS_0_ (285 CNY/Ton), and 34.8% lower than 42.5 Portland cement (350 CNY/Ton), despite achieving comparable mechanical performance. This cost reduction stemmed predominantly from replacing high-cost cement with low-value solid wastes, underscoring the economic viability of SAC-PHS for sustainable construction.

Moreover, PHS_25_ exhibited dual advantages: (1) reduced embodied carbon and material costs relative to both PHS_0_ and 42.5 Portland cement, and (2) maintained competitive mechanical properties. Given that its raw materials consist largely of industrial solid waste, this system offers notable environmental benefits and promising economic potential for large-scale applications.

## 4. Conclusions

This study successfully synthesized SAC-PHS using phosphorous slag, ground granulated blast furnace slag, Portland cement, fly ash, and desulfurization gypsum. Key conclusions include:

(1) Phosphorous slag could be used as a supplementary cementitious material to prepared silica-aluminum based cementitious materials (SAC-PHS). The PHS_25_ with good mechanical properties was prepared by mixing 25 wt% phosphorous slag, 40 wt% ground granulated blast furnace slag (GBFS), 20 wt% PII 52.5 cement, 10 wt% fly ash, and 5 wt% desulfurization gypsum. The flexural and compressive strengths of PHS_25_ at 7 days attained 9.0 MPa and 36.5 MPa, respectively.
(2) The one-week hydration products of SAC-PHS mainly included C-A-S-H gels, Ca(OH)_2_, and ettringite (AFt). C-A-S-H gels and AFt played an active role in the development of one-week strength. The C-A-S-H gels and AFt produced by SAC-PHS when the content of phosphorous slag reached 25% (PHS_25_) were the bulk, leading to a smaller pore size and more uniform pore distribution. The P/Si atomic ratio showed that during the formation of C-A-S-H gels, a bond was formed between Si and P, which improved the strength of the SAC-PHS material.
(3) The aluminum in the one-week hydration products of SAC-PHS existed in the form of four and six coordination, proving that the four-coordination Al in the raw material participated in the hydration reaction to form C-A-S-H gels and AFt. The [SiO_4_] polymerization of C-A-S-H gels was mainly in the form of SiQ^2^(1Al) in one-week hydration time.
(4) The embodied carbon of PHS_25_ was 0.22 kgCO_2_/kg, which is 74.12% lower than the embodied carbon of 42.5 Portland cement and 37.14% lower than that of PHS_0_. The material cost of PHS_25_ was 228 CNY/Ton, which is 20.0% lower than PHS_0_ and 34.8% lower than 42.5 Portland cement, presenting significant environmental and ecological values.

## Figures and Tables

**Table 1:** Origin of raw materials.

Raw Material	Origin of Raw Material
Phosphorous slag	Guizhou Phosphorous Slag, Guiyang, China
Ground granulated blast furnace slag	Tangshan Iron and Steel Group, Tangshan, China
PII 52.5 Portland cement	Anhui Conch Cement, Wuhu, China
Fly ash	Shandong Weiqiao Group, Binzhou, China
Desulfurization gypsum	Shandong Weiqiao Group, Binzhou, China
Standard sand	Xiamen ISO Standard Sand Co., Ltd., Xiamen, China

**Table 2:** Experimental proportioning of silica-alumina based cementitious materials composed of phosphorous slag.

No.	PHS /wt%	GBFS/wt%	PII 52.5 Portland Cement/wt%	Fly Ash/wt%	Desulfurization Gypsum/wt%
PHS_0_	0	50	36	10	4
PHS_20_	20	45	20	10	5
PHS_25_	25	40	20	10	5
PHS_30_	30	35	20	10	5
PHS_35_	35	30	20	10	5
PHS_40_	40	25	20	10	5

**Table 3:** Analysis results of the ^27^Al-NMR spectra for PHS_20_, PHS_25_, and PHS_30_.

Sample		Al^IV^	Al^VI^
	Chemical Shift (ppm)	66.5	9.5–13.3
PHS_20_	Relative area	100	69.7
	Relative content (%)	58.9	41.1
	Chemical Shift (ppm)	63.4	9.5–13.3
PHS_25_	Relative area	100	89.8
	Relative content (%)	52.7	47.3
	Chemical Shift (ppm)	65.4	9.5–13.3
PHS_30_	Relative area	100	74.7
	Relative content (%)	57.2	42.7

**Table 4:** Analysis results of the ^29^Si-NMR spectra for PHS_20_, PHS_25_, and PHS_30_.

Sample	Chemical Shift (ppm)	Type	Relative Area	RBO
PHS_20_	−73.9	SiQ^0^	18.8	50.0%
	−83.1	SiQ^2^(1Al)	100	
	−99.9	SiQ^3^	10.6	
	−111.7	SiQ^4^	12.9	
PHS_25_	−74.3	SiQ^0^	16.1	51.0%
	−82.6	SiQ^2^(1Al)	100	
	−93.9	SiQ^3^	22.8	
	−111.6	SiQ^4^	8.6	
PHS_30_	−73.9	SiQ^0^	16.4	45.0%
	−83.0	SiQ^2^(1Al)	100	
	−98.7	SiQ^3^	4.5	
	−111.3	SiQ^4^	0.8	

**Table 5:** Embodied carbon data and market prices for raw materials.

Material	Embodied Carbon	Market Price
	(kgCO_2_/kg)	(CNY/Ton)
PHS	0.05	100
GBFS	0.0416	300
PII 52.5 Cement	0.912	330
Fly ash	0.01	150
Desulfurization gypsum	0.12	30
42.5 Portland Cement	0.85	350

## Data Availability

The original contributions presented in this study are included in the article. Further inquiries can be directed to the corresponding authors.
